# Influence of Secondary Hyperparathyroidism Induced by Low Dietary Calcium, Vitamin D Deficiency, and Renal Failure on Circulating Rat PTH Molecular Forms

**DOI:** 10.1155/2011/469783

**Published:** 2011-06-22

**Authors:** Pierre D'Amour, Louise Rousseau, Stephen Hornyak, Zan Yang, Tom Cantor

**Affiliations:** ^1^Centre de Recherche, Centre Hospitalier de l'Université de Montréal (CRCHUM), Hôpital Saint-Luc Département de Médecine, Université de Montréal, Montréal, QC, Canada H2X 1P1; ^2^Scantibodies Laboratory Inc., Santee, CA 92071, USA

## Abstract

Rats(r) with secondary hyperparathyroidism were studied to define the relationship between vitamin D metabolites and rPTH levels measured by 3 different rat ELISAs. Controls and renal failure (RF) rats were on a normal diet, while 2 groups on a low-calcium (-Ca) or a vitamin D-deficient (-D) diet. RF was induced surgically. Mild RF rats had normal calcium and 25(OH)D but reduced 1,25(OH)_2_D levels (*P* < .001) with a 2.5-fold increased in rPTH (*P* < .001). Severe RF rats and those on a -Ca or -D diet had reduced calcium (*P* < .01) and 25(OH)D levels (*P* < .05), with rPTH increased by 2 (-Ca diet; *P* < .05), 4 (-D diet; *P* < .001), and 20-folds (RF; *P* < .001) while 1,25(OH)_2_D was high (-Ca diet: *P* < .001) or low (-D diet, RF: *P* < .001). 25(OH)D and 1,25(OH)_2_D were positively and negatively related on the -Ca and -D diets, respectively. rPTH molecular forms behaved as expected in RF and on -Ca diet, but not on -D diet with more C-rPTH fragments when less were expected. This may be related to the short-time course of this study compared to prior studies.

## 1. Introduction

The behavior of circulating PTH molecular forms during the development of secondary hyperparathyroidism (sHPT) is related to the cause of sHPT. In experimental calcium and vitamin D-deficient dogs, ionized calcium remains unchanged until 1,25(OH)_2_D starts to decline after an initial increment. This behavior was attributed to 25(OH)D levels dipping below 20 nmol/L [1]. Furthermore, as sHPT progresses, basal, stimulated, and nonsuppressible Intact (I) PTH increases more than carboxyl-terminal (C) PTH levels, indicating a shift in PTH secretion toward PTH(1-84) [1]. When these dogs were replenished with vitamin D, ionized calcium and 25(OH)D normalized rapidly while 1,25(OH)_2_D initially reached supraphysiological values which progressively regressed to normal with time [2]. While I-PTH normalized to prior vitamin D deficiency values, C-PTH remained elevated with a 2-fold rise in the C-PTH/I-PTH ratio, indicating adaptation to increased parathyroid tissue mass through oversecretion of C-PTH fragments [2]. The latter finding was further sustained by low-dose 1,25(OH)_2_D therapy in normal dogs which, without augmenting calcium concentration, caused a decrease in I-PTH but not C-PTH secretion, heightening the C-PTH/I-PTH ratio at all calcium concentrations [3]. This tight regulation of C-PTH fragments suggested possible biological effects of these fragments which were eventually demonstrated both in vivo [4–6] and in vitro [7]. C-PTH fragments exert biological effects which are opposite to those of PTH(1-84) or PTH(1-34) on the PTH/PTHrP type I receptor by acting on a different receptor [4–7]. The evolution of circulating PTH molecular forms during sHPT due to renal failure (RF) is different, mainly because C-PTH fragments are cleared by the kidneys [8–10]. Hypocalcemia usually develops when the glomerular filtration rate (GFR) falls below 30 ml/min/1.73 m^2^ in relation to low 1,25(OH)_2_D and high phosphate levels [10]. The elevation of I- and C-PTH levels is rapid and already evident in stage 2 disease (GFR >60 ml/min/1.73 m^2^) with more rapid progression of C-PTH than I-PTH values as the GFR decreases [10]. RF patients with severe sHPT manifest a lower C-PTH/I-PTH ratio than patients with mild sHPT, indicating higher PTH(1-84) production relative to C-PTH fragments in severe sHPT [8]. 25(OH)D levels also decrease as GFR decreases [11–15] but the mecanisms involved are unclear. Rat(r) PTH ELISAs developed by Scantibodies Laboratory Inc. were used to study the relationship between rPTH molecular forms, 25(OH)D and 1,25(OH)_2_D concentrations, during sHPT ascribed to a low-calcium diet (-Ca diet), a vitamin D-deficient diet (-D diet), or RF. With some minor differences, results indicate a similar behavior of circulating PTH molecular forms in various species.

## 2. Materials and Methods

### 2.1. Experimental Methods

#### 2.1.1. Products

All special diets were purchased from Harlan Teklad (Madison, WI, USA). All hPTH and rPTH peptides were obtained from BACHEM (Torrance, CA, USA), some from the regular catalog and others newly synthesized for this project.

#### 2.1.2. Biochemistry

Ionized calcium (Ca^2+^) was quantitated by Ca^2+^-specific electrode (Rapid Lab Model 348, Bayer Diagnostic, Toronto, ON, Canada). Total calcium, phosphate, albumin, creatinine, and alkaline phosphatase were measured by colorimetric methods adapted to multianalyzer evaluation. For other measurements, samples remained at room temperature for a maximum of 30 minutes and were transferred and centrifuged at 4°C within 2 hours, aliquoted and stored at −80°C until assayed. Serum was used for all assays. 25(OH)D was studied by a radioimmunoassay from IDS Inc. (Fountain Hills, AZ, USA). 1,25(OH)_2_D was assessed by enzyme-linked immune-sorbent assay (ELISA) from ALPCO Diagnostics (Salem, NH, USA). rPTH was investigated by 3 different ELISAs from Scantibodies Laboratory Inc. (Santee, CA, USA). The characteristics of the three rPTH ELISAs have been described previously [16].

#### 2.1.3. HPLC Analysis

Sera were pooled from all animals in each group for HPLC analysis of circulating rPTH molecular forms. Each pool of sera was first extracted with Waters Sep-Pak Plus C-18 cartridges, as described by Bennett et al. [17]. One C-18 cartridge was employed for each 3 ml of serum. Samples were eluted from the cartridge with 5 ml of 800 ml/L acetonitrile in 1 g/L trifluoroacetic acid. Acetonitrile was evaporated from the eluate with nitrogen, and the residual volume was freeze-dried and reconstituted in 0.5 ml of 1 g/L triofluoroacetic acid for HPLC analysis. Each 0.5 mL sample was loaded on a Waters C_18_ *μ*Bondapak analytical column (300 × 3.9 mm (i.d.)) and eluted with a noncontinuous linear gradient of acetonitrile in 1 g/L trifluoroacetic acid. The gradient, ranging from 19 to 33.5% in 15 minutes and from 33.5 to 35.2% in 45 minutes, was delivered at 1.0 mL/min with an Agilent 1100 series solvent delivery system. 1.0 mL fractions were evaporated, freeze-dried, and reconstituted to 1 mL with 7 g/L BSA in water; adequate volumes were then measured in each rPTH assay. Control experiments were performed with rPTH(1-84) calibrator added to hypoparathyroid rat serum to ensure that PTH degradation did not occur during the various procedures. A single peak of immunoreactivity coeluting with rPTH(1-84) was detected by the 3 rPTH assays. Immunoreactive rPTH recovery by rPTH assays through all these procedures was better than 80% for all HPLC runs, based on comparison of the original pool rPTH value with the sum of rPTH immunoreactivity across all HPLC fractions.

#### 2.1.4. Experimental Animals

Male Sprague-Dawley rats were purchased at 76 to 100 g from Charles-River Canada (St-Constant QC, Canada) and maintained in cages according to the guidelines of the Canadian Council on Animal Care. Three groups (1 control and 2 renal failure (RF) groups) were maintained on a normal calcium and vitamin D diet as soon as they were delivered to our centre. Two groups were fed either a low-calcium (0.1% calcium 0.3% phosphorus) vitamin D-sufficient or vitamin D-deficient calcium sufficient (0.5% calcium and 0.3% phosphorus) diet. All groups received their respective diets for 33 days. RF was induced by 5/6 nephrectomy under general anesthesia as 2 consecutive procedures, 2/3 nephrectomy of the left kidney on the first day, followed by right total nephrectomy 6 days later. Eventually, these rats were divided into 2 groups according to a serum creatinine value below or above 150 *μ*mol/L. They were sacrificed after 33 days of diet or 33 days after the second surgery. In all cases, they were sacrificed under general anesthesia by exsanguination through the abdominal vena cava.

#### 2.1.5. Statistical Analysis

The results are means ± SD. Differences between groups were analyzed by Kruskal-Wallis ANOVA, followed by Dunn's multiple comparison test when the distribution of values was not Gaussian or by ANOVA, followed by Dunnett's multiple comparison test when the distribution was Gaussian. -Ca diet and -D diet rats and the 2 RF groups were compared by unpaired T-test with or without Welch correction. Standard methods were used for linear regression between various biochemical parameters. HPLC profiles were evaluated planimetrically by Origin 8.5 software (OriginLab Corporation, Northampton, MA, USA). HPLC profiles were corrected to 100% recovery and to the mean pool value expressed in pmol/L.

## 3. Results

**Figure 1:**
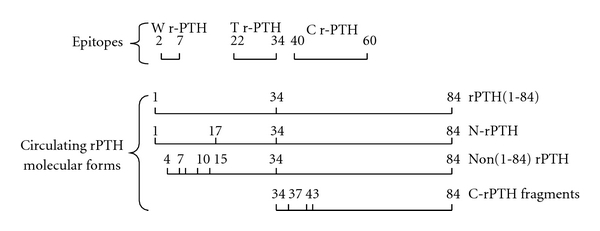
Relationship between rPTH assays epitopes and circulating rPTH molecular forms detected. N-PTH = amino-terminal rPTH, a posttranslationally modified form of rPTH(1-84) on serine 17. Non(1-84) large C-rPTH fragments have a partially preserved N-structure while smaller C-rPTH fragments miss that structure (18).

Figure 1 illustrates the relationship between rPTH assays epitopes and detected circulating rPTH molecular forms. The Whole (W) rPTH assay has a 2–7 epitope and reacts with rPTH(1-84) and N-rPTH, a posttranslationaly modified form of rPTH(1-84) on circulating rPTH HPLC profiles. The Total (T) rPTH assay has a 22–34 epitope and reacts with rPTH(1-84), N-rPTH, and non-(1-84) rPTH or large carboxyl-terminal (C) fragments with a partially preserved N-structure, the prototype of which is rPTH(7-84). The C-rPTH assay has a 40–60 epitope and reacts with rPTH(1-84), N-rPTH, non-(1-84) rPTH, but mainly with smaller C-rPTH fragments missing an N-structure which represent the majority of circulating rPTH. The C-rPTH assay has a greater affinity for these fragments than for the other circulating PTH molecular forms.

**Figure 2:**
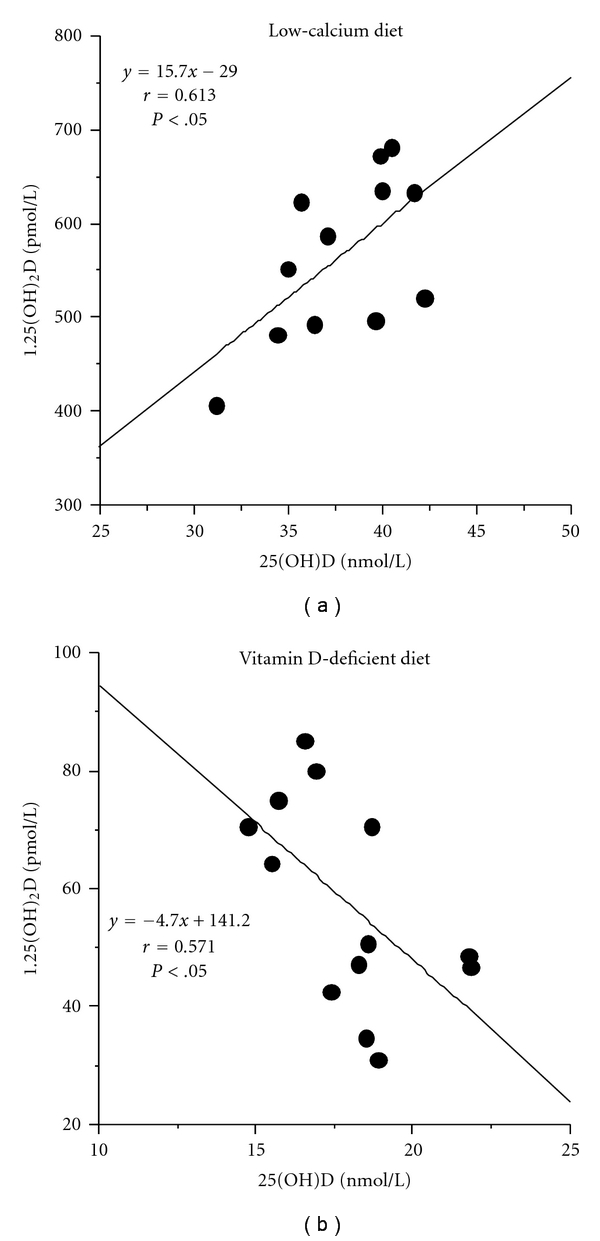
Relationship between 25(OH)D and 1,25(OH)_2_D levels in rats on the calcium-deficient diet (a) and the vitamin D-deficient diet (b).

**Figure 3:**
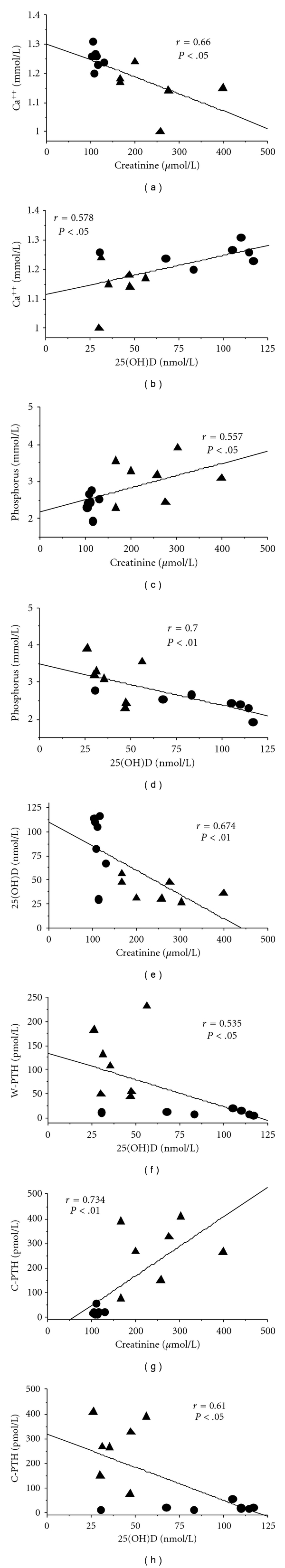
Relationship between creatinine or 25(OH)D and calcium, phosphate, and rPTH levels in rats with moderate (●) or severe (▲) renal failure.

Compared to the controls, rats on a -Ca diet (Tables 1 and 2) had decreased total calcium concentration (*P* < .01), 2-fold increased alkaline phosphatase activity (*P* < .001), low 25(OH)D (*P* < .05) but elevated 1,25(OH)_2_D levels (*P* < .001), and a greater increment of C-rPTH (*P* < .001) than W-rPTH (*P* < .05). C-rPTH/W-rPTH and C-rPTH/T-rPTH ratios tended to rise without reaching significance. A positive correlation was observed between 25(OH)D and 1,25(OH)_2_D levels (*P* < .05) on the -Ca diet (Figure 2(a)). Rats on the -D diet (Tables 1 and 2) had low ionized and total calcium values (*P* < .01), low 25(OH)D (*P* < .001) and 1,25(OH)_2_D (*P* < .001), a 2- to 3-fold elevation of rPTH (*P* < .001), and a significant increase in the C-rPTH/T-rPTH ratio (*P* < .05). Their 25(OH)D and 1,25(OH)_2_D levels were lower (*P* < .001) and their rPTH levels were higher (*P* < .01) than those in rats on a -Ca diet. A negative correlation was noted between 25(OH)D and 1,25(OH)_2_D levels (*P* < .05) on the -D diet (Figure 2(b)). Rats with moderate RF (Tables 1 and 2) remained normocalcemic with a 3-fold rise in serum creatinine, maintained normal 25(OH)D with low 1,25(OH)_2_D levels (*P* < .001), and presented 2- to 3-fold higher W-rPTH (*P* < .001) and C-rPTH (*P* < .001) levels. Their T/W-rPTH was significantly decreased (*P* < .05). Finally, rats with severe RF (Tables 1 and 2) had low body weight (*P* < .01), low ionized calcium (*P* < .001), an 8-fold increase in serum creatinine (*P* < .001), diminished alkaline phosphatase activity (*P* < .001), reduced 25(OH)D (*P* < .05) and 1,25(OH)_2_D levels (*P* < .001), and a 20- to 35-fold elevation of W (*P* < .001), T (*P* < .001), and C-rPTH (*P* < .001) with diminution of the T-rPTH/W-rPTH ratio (*P* < .001) and a heightened C-rPTH/T-rPTH ratio (*P* < .001). Their 25(OH)D levels were lower (*P* < .01) and rPTH levels (*P* < .05) were much higher than those in rats with moderate RF. A negative correlation was apparent between ionized calcium, 25(OH)D and creatinine levels (*P* < .05), with a positive correlation between phosphate, C-rPTH levels, and creatinine concentrations (*P* < .05) (Figure 3). A positive correlation was found between ionized calcium and 25(OH)D levels (*P* < .05) and negative correlations between 25(OH)D and phosphate or W or C-rPTH levels (*P* < .05) (Figure 3).

**Figure 4:**
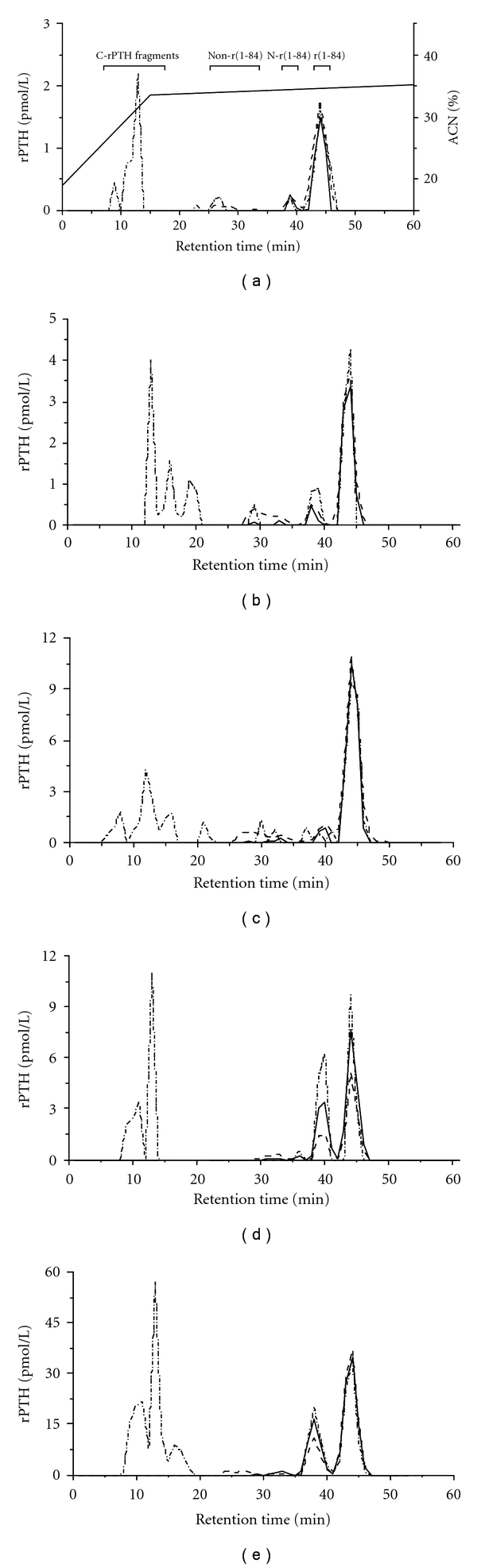
HPLC profiles of circulating rPTH molecular forms analyzed in Whole (–), Total (- - - -), and Carboxyl-rPTH (-.-.-.-) assays in rats on a normal calcium diet (a), a low-calcium diet (b), a vitamin D-deficient diet (c), or with moderate (d) or severe (e) renal failure. A single HPLC profile obtained with a pool of serum from all rats in a given group is illustrated.

Figure 4 and Table 3 summarize the results of high-pressure liquid chromatography (HPLC) profile analysis in the various groups. Rats on a normal calcium diet, like the other groups, disclosed 4 types of rPTH molecular forms in the circulation: rPTH(1-84) (regions 42 to 46), N-rPTH (regions 38 to 42), a posttranslationally modified form of rPTH(1-84), non-(1-84) rPTH fragments (regions 25–32) or large C-PTH fragments with a partially preserved N-structure, and C-rPTH fragments (regions 8 to 20) which lacked an N-structure. rPTH(1-84) was identified by all 3 rPTH assays, as was N-rPTH. N-rPTH tended to be over-evaluated by C-rPTH assay in relation to the use of a rPTH(39-84) standard which was more immunoreactive on a molar basis than the rPTH(1-84) standard. Non-(1-84) PTH fragments were mainly detected by T-rPTH assay, and smaller C-rPTH fragments exclusively by C-rPTH assay. With W-rPTH assay, rPTH(1-84) represented more than 90% of immunoreactivity in the diet groups, but only 66% in RF rats relative to N-rPTH accumulation. With T-rPTH assay, all groups had lower % rPTH(1-84) than the controls. This was attributed to more non-(1-84) rPTH fragments on the -Ca and -D diets and to more N-rPTH in RF. Finally, with C-rPTH assay, rPTH(1-84) represented 46 and 39% of rPTH in control and -Ca diet rats and 52% in -D diet rats. These percentages decreased to 29 and 27% in moderate and severe RF rats, respectively, in relation to N-rPTH accumulation in moderate RF and C-rPTH fragments in severe RF. In each case, C-rPTH fragments explained 34 to 57% of non-rPTH(1-84) immunoreactivity with the highest % in RF rats.

## 4. Discussion

The 3 rPTH assays used in this study were demonstrated to react with rPTH molecular forms similarly to those detected in humans [18–21] and dogs [3]. Thus, rPTH(1-84), N-rPTH, non-(1-84) rPTH, and C-rPTH fragments were noted on rat HPLC profiles. Differences from hPTH HPLC profiles may include more rPTH(1-84) and less C-rPTH fragments in rats compared to humans in the basal state [20]. 

These assays were tested to investigate the behavior of circulating rPTH molecular forms in sHPT induced by a -Ca diet, a -D diet, and RF induced by 5/6 nephrectomy. With the -Ca diet, we observed decreased total calcium levels, increased alkaline phosphatase activity, low 25(OH)D with very high 1,25(OH)_2_D concentrations, and elevated W- and C-rPTH levels. A positive correlation was seen between 25(OH)D and 1,25(OH)_2_D levels. rPTH ratios remained unchanged. Diminished intestinal absorption of calcium due to very low calcium supply, even if 1,25(OH)_2_D concentrations were augmented, best explained the hypocalcemia [22, 23]. The high alkaline phosphatase activity was attributed to a defective mineralization rate in the face of elevated 1,25(OH)_2_D concentrations [24, 25]. The decreased 25(OH)D concentration was probably due to heightened circulating levels of 1,25(OH)_2_D which enhanced 25(OH)D turnover into inactive metabolites [26, 27]. C-rPTH levels rose more than T- and W-rPTH levels. More non-(1-84) rPTH was found by T-rPTH assay, and C-rPTH fragments by C-rPTH assay on HPLC profiles, a surprising observation as a greater proportion of rPTH(1-84) might have been expected [28]. An enhanced turnover of rPTH(1-84) into non-(1-84) rPTH and C-rPTH fragments appears to be responsible and may be ascribed to elevated 1,25(OH)_2_D concentrations [3, 29]. Despite heightened 1,25(OH)_2_D levels, rPTH concentrations increased but less than in -D diet rats. This is likely the result of a partial inhibitory effect of calreticulin on 1,25(OH)_2_D binding to its receptor in the parathyroid glands in the presence of hypocalcemia and sHPT [30, 31].

A slightly different situation prevailed in rats maintained on a -D diet for 33 days. The calcium level was decreased with normal phosphate concentration, normal alkaline phosphatase activity, low 25(OH)D and 1,25(OH)_2_D concentrations, elevated rPTH levels, and a high C/T-rPTH ratio. A negative correlation was observed between 25(OH)D and 1,25(OH)_2_D levels. Hypocalcemia was best explained by reduced intestinal absorption of calcium induced by low 1,25(OH)_2_D [32, 33] in relation to very low substrate concentrations [1]. The latter is probably the consequence of deficient vitamin D supply [1]. rPTH levels were elevated because of hypocalcemia and low 1,25(OH)_2_D, both of which stimulated rPTH production via different mechanisms [34–36]. C/T-rPTH rose because of greater C-rPTH than T-rPTH increment possibly due to the catabolism of secreted rPTH(1-84) into C-rPTH fragments. This differs from our dog model of D deficiency where % PTH(1-84) was increased [1, 28], but the latter study was of two-year duration. A slightly longer study might have provided a calcemic dissociation between -Ca and -D diets. In dogs after 3 weeks of a -Ca -D diet, C-PTH fragments were also elevated (28). 

In moderate RF, a 3-fold augmentation of serum creatinine was observed with normal calcium level, normal 25(OH)D and low 1,25(OH)_2_D concentrations, increased W- and C-rPTH levels, and a decreased T/W-rPTH ratio. Two-thirds of the rPTH elevation was explained by rPTH(1-84) and one-third by N-rPTH on W-rPTH assay. The % of C-rPTH fragments was not enhanced in C-rPTH assay, but this may be related to overevaluation of N-PTH associated with the use of rPTH(39-84) standard. Low 1,25(OH)_2_D levels linked with RF [37] contributed to the decrease in intestinal calcium absorption [32, 33], while increased rPTH(1-84) levels contributed to normal phosphate levels [38]. Since W-rPTH increased more than T-rPTH, the T/W-rPTH ratio declined mostly in relation to more N-rPTH detected by W-rPTH assay. In more severe RF, rats had impaired growth with lower body weight [39–41]. Serum creatinine was increased by 8-fold, hypocalcemia was present with normal phosphate levels, and alkaline phosphatase activity was decreased, as were 25(OH)D and 1,25(OH)_2_D levels, and W- and T-rPTH were augmented 30- and 20-fold, respectively, with C-rPTH more than 35-fold. The T/W-rPTH ratio was reduced and the C/T-rPTH ratio increased. Hypocalcemia was best explained again by low intestinal calcium absorption ascribed to low 1,25(OH)_2_D [32, 33]. The phosphate level was still normal because rPTH was augmented, and RF was still not significantly sufficient to impair renal phosphate excretion [38], but the phosphate level was more elevated than in moderate RF. The decrease in alkaline phosphatase activity reflected the much lower weight of these rats compared to the other groups, and their impaired growth was related to advanced RF [39–41]. Low 25(OH)D levels could not be linked to high 1,25(OH)_2_D levels in these rats. Decreased intestinal vitamin D absorption [42] in part related to their lower weight and eating profile and reduced 25(OH)D production associated with cytochrome P450 diminution in the liver by very high rPTH levels [43] may have been responsible. As in rats with milder RF, W-rPTH elevation was two-thirds related to rPTH(1-84) and one-third to N-PTH. Accumulation of C-rPTH fragments was observed with more advanced RF, as in humans [9]. The decreased T/W-rPTH ratio is explained by the same mechanism seen in moderate RF, while the increased C/T-rPTH ratio is attributed to the accumulation of C-rPTH fragments.

In summary, our results indicate that rPTH assays with characteristics similar to those in hPTH assays are useful tools to identify rPTH molecular forms similar to those found in humans. Quantitative differences may exist with humans in relation to more rPTH(1-84) and less C-rPTH fragments in rats. In models of sHPT due to a -Ca diet, a -D diet, or severe RF, we discerned low 25(OH)D levels. If decreased vitamin D supply on the -D diet and elevated 25(OH)D turnover induced by high 1,25(OH)_2_D levels on the -Ca diet explain the low 25(OH)D level, they could not explain the low 25(OH)D level in RF. Reduced intestinal absorption of vitamin D [42] and diminished hepatic 25(OH)D synthesis related to a direct effect of severe sHPT on cytochrome P450 level in the liver [43] may be implicated. More studies will be required to investigate these mechanisms.

The behavior of rPTH molecular forms derived from rPTH ratios was as expected in severe RF with the accumulation of smaller C-rPTH fragments and an increase in the C/T-rPTH ratio. This is believed to contribute to the PTH resistance observed in renal failure experimentally (4–7). Similarly, the increase in smaller C-rPTH fragments on the -Ca diet was expected even if C/T and C/W-rPTH ratios were not significantly increase in relation with the very high levels of 1,25(OH)2D which will influence the degradation of PTH(1-84) into fragments in the parathyroid glands (3, 29). The only unexplained situation remains the increase in C-rPTH fragments on the -D diet with the increase C/T-rPTH ratio when one would have expected more rPTH(1-84) as in a dog model (1). The short-time course of rat experiments may be involved in these differences because on a -Ca -D diet in dogs a similar profile was observed at 3 weeks (28). Overall this study demonstrates a behavior of rPTH molecular forms similar to other species with minor differences related to the shorter-time course of the study.

##  Disclosure Statement

This work was made possible through a grant from Scantibodies Laboratory Inc. to the main investigator, Pierre D'Amour. T. Cantor is the President of Scantibodies Laboratory Inc. and S. Hornyak and Z. Yang are employees of the same company. L. Rousseau has no disclosure.

## Figures and Tables

**Table 1:** Evolution of biochemical parameters with various diets and partial nephrectomy.

Parameters	Normal calcium (0.5%)	Low calcium (0.1%)	Vitamin D deficiency	Moderate renal failure	Severe renal failure
N	10	12	13	7	7
Weight (g)	405 ± 31	379 ± 35	390 ± 30	374 ± 45	278 ± 64^∗∗∗,••^
Ca^++^ (mmol/L)	1.30 ± 0.02	1.28 ± 0.03	1.24 ± 0.02^∗∗,++^	1.25 ± 0.03	1.15 ± 0.08^∗∗∗,•^
Ca_t_ (mmol/L)	2.58 ± 0.06	2.42 ± 0.10**	2.42 ± 0.09**	2.56 ± 0.07	2.47 ± 0.20
PO_4_ (mmol/L)	3.01 ± 0.27	3.15 ± 0.11	2.86 ± 0.27^++^	2.44 ± 0.27**	3.10 ± 0.57^•^
Creatinine (*μ*mol/L)	30 ± 2	31 ± 4	34 ± 7	111 ± 9**	252 ± 84^∗∗∗,••^
Alkaline phosphatase (U/L)	403 ± 138	939 ± 372***	351 ± 177^+++^	259 ± 56	174 ± 62^∗∗∗,•^
Total protein (g/L)	52 ± 2	54 ± 2	55 ± 2	51 ± 2	45 ± 6^•^
Albumin (g/L)	23 ± 1	23 ± 1	24 ± 1^+^	21 ± 1**	20 ± 1^∗∗∗,•^
25(OH)D (nmol/L)	97 ± 18	38 ± 3*	18 ± 2^∗∗∗,+++^	90 ± 32	39 ± 11^∗,••^
1,25(OH)_2_D (pmol/L)	129 ± 51	565 ± 86***	58 ± 18^∗∗∗,+++^	46 ± 10***	38 ± 8***

Results are means ± SD. All group statistics were assessed by ANOVA, followed by Dunnett's multiple comparison test (Kruskal-Wallis ANOVA, followed by Dunn's comparison test for creatinine, total protein, and 25(OH)D). *P < .05; **P < .01; ***P < .001. Unpaired T-test with or without Welch correction: Low-calcium versus vitamin D-deficient diet: ^+^ *P* < .05; ^++^ *P* < .01; ^+++^ *P* < .001. Moderate versus severe renal failure: ^•^ *P* < .05; ^••^ *P* < .01; ^•••^ *P* < .001.

**Table 2:** Evolution of rPTH and rPTH ratio with various diets and partial nephrectomy.

Parameters	Normal calcium (0.5%)	Low calcium (0.1%)	Vitamin D deficiency	Moderate renal failure	Severe renal failure
N	10	12	13	7	7
W-rTH (pmol/L)	4.0 ± 1.2	7.7 ± 3.9*	15.0 ± 7.6^∗∗∗,++^	11.6 ± 4.9***	114 ± 72.6^∗∗∗,••^
T-rPTH (pmol/L)	5.9 ± 1.4	11.1 ± 5.1	20.8 ± 9.9^∗∗∗,++^	13.4 ± 6.9	116.1 ± 79.1^∗∗∗,•^
C-rPTH (pmol/L)	7.3 ± 2.6	17.4 ± 8.9***	37.2 ± 15.8^∗∗∗,+++^	23.1 ± 15.0***	268.4 ± 121.4^∗∗∗,••^
T/W-rPTH ratio	1.50 ± 0.13	1.48 ± 0.12	1.41 ± 0.10	1.12 ± 0.38*	0.97 ± 0.13***
C/W-rPTH ratio	1.85 ± 0.46	2.35 ± 0.47	2.66 ± 0.70	2.02 ± 0.84	2.73 ± 1.51
C/T-rPTH ratio	1.24 ± 0.35	1.58 ± 0.29	1.88 ± 0.49*	2.24 ± 1.65	2.96 ± 2.07***

Results are means + SD. All group statistics were assessed by ANOVA, followed by Dunnett's multiple comparison test (Kruskal-Wallis ANOVA followed by Dunn's comparison test for rTotal-PTH, rTotal/rWhole ratio, rCarboxyl/rTotal ratio) *P < .05; **P < .01; ***P < .001. Unpaired T-test with or without Welch correction: low-calcium versus vitamin D-deficient diet: ^+^ *P* < .05; ^++^ *P* < .01; ^+++^ *P* < .001. Moderate versus severe renal failure: ^•^ *P* < .05; ^••^ *P* < .01; ^•••^ *P* < .001.

**Table 3:** Analysis of rPTH HPLC profiles obtained by each rPTH assay.

Groups	rPTH assay	Pool value (pmol/L)	Rec. (%)	C-PTH fragments (pmol/L) (%)	Non(1-84) PTH	N-PTH	PTH(1-84)
Normal-Ca diet	Whole	3.6	117		0 (0.4)	0.3 (8.5)	3.3 (91.1)
	Total	5.4	99		0.3 (5.8)	0.4 (8.2)	4.6 (86.0)
	C	9.0	97	4.2 (46.6)	0.5 (5.2)	0.2 (2.2)	4.1 (46.0)

Low-Ca diet	Whole	7.6	111		0.2 (2.1)	0.5 (7.1)	6.9 (90.8)
	Total	11.1	94		1.7 (15.6)	0.9 (8.3)	8.4 (76.1)
	C	17.7	95	8.5 (48.3)	0.5 (2.8)	1.7 (9.6)	7.0 (39.3)

Vitamin D-deficient diet	Whole	25.5	108		0.4 (1.6)	1.5 (6.0)	23.6 (92.4)
	Total	34.0	103		3.9 (11.4)	2.7 (7.9)	27.4 (80.7)
	C	45.0	116	15.5 (34.5)	3.5 (7.7)	2.4 (5.3)	23.6 (52.5)

Moderate renal failure	Whole	22.8	114		0.4 (1.9)	7.4 (32.6)	14.9 (65.5)
	Total	15.0	108		1.3 (8.7)	3.5 (23.1)	10.2 (68.2)
	C	43.2	100	18.9 (43.8)	0.5 (1.2)	11.1 (25.8)	12.6 (29.2)

Severe renal failure	Whole	127.3	99		2.8 (2.2)	38.6 (30.3)	85.9 (67.5)
	Total	122.0	83		5.7 (4.7)	24.6 (20.2)	91.6 (75.1)
	C	278.0	107	157.9 (56.8)	0.0 (0.0)	45.0 (16.2)	75.1 (27.0)

Results of 1 HPLC profile in each case.
